# From SARS and MERS to COVID-19: a brief summary and comparison of severe acute respiratory infections caused by three highly pathogenic human coronaviruses

**DOI:** 10.1186/s12931-020-01479-w

**Published:** 2020-08-27

**Authors:** Zhixing Zhu, Xihua Lian, Xiaoshan Su, Weijing Wu, Giuseppe A. Marraro, Yiming Zeng

**Affiliations:** 1 Department of Pulmonary and Critical Care Medicine, the Second Affiliated Hospital of Fujian Medical University, Respirology Medicine Centre of Fujian Province, 34 Zhongshanbei Road, Licheng District, Quanzhou, China; 2 Department of Ultrasound Medicine, the Second Affiliated Hospital of Fujian Medical University, 34 Zhongshanbei Road, Licheng District, Quanzhou, China; 3 Healthcare Accountability Lab, University of Milan, Via Festa Del Perdono, Milan, Italy

**Keywords:** SARS-CoV, MERS-CoV, SARS-CoV-2, Illness, Biological features, Clinical characteristics

## Abstract

Within two decades, there have emerged three highly pathogenic and deadly human coronaviruses, namely SARS-CoV, MERS-CoV and SARS-CoV-2. The economic burden and health threats caused by these coronaviruses are extremely dreadful and getting more serious as the increasing number of global infections and attributed deaths of SARS-CoV-2 and MERS-CoV. Unfortunately, specific medical countermeasures for these hCoVs remain absent. Moreover, the fast spread of misinformation about the ongoing SARS-CoV-2 pandemic uniquely places the virus alongside an annoying infodemic and causes unnecessary worldwide panic. SARS-CoV-2 shares many similarities with SARS-CoV and MERS-CoV, certainly, obvious differences exist as well. Lessons learnt from SARS-CoV and MERS-CoV, timely updated information of SARS-CoV-2 and MERS-CoV, and summarized specific knowledge of these hCoVs are extremely invaluable for effectively and efficiently contain the outbreak of SARS-CoV-2 and MERS-CoV. By gaining a deeper understanding of hCoVs and the illnesses caused by them, we can bridge knowledge gaps, provide cultural weapons for fighting and controling the spread of MERS-CoV and SARS-CoV-2, and prepare effective and robust defense lines against hCoVs that may emerge or reemerge in the future. To this end, the state-of-the-art knowledge and comparing the biological features of these lethal hCoVs and the clinical characteristics of illnesses caused by them are systematically summarized in the review.

## Background

Coronaviruses (CoVs) refer to a family of enveloped, positive-sense, single-stranded, and highly diverse RNA viruses [1]. There are four genera (alpha, beta, gamma, and delta), among which α-coronavirus and β-coronavirus attract more attention because of their ability to cross animal-human barriers and emerge to become major human pathogens [2]. So far, there are seven documented human coronaviruses (hCoVs), including the beta-genera CoVs, namely Severe Acute Respiratory Syndrome (SARS)-CoV (SARS-CoV), Middle East Respiratory Syndrome (MERS)-CoV (MERS-CoV), SARS-CoV hCoV-HKU1, and hCoV-OC43 and the α-genera CoVs, which are hCoV-NL63 and hCoV-229E, respectively [1, 3].

Although hCoV-HKU1, hCoV-OC43, hCoV-NL63 and hCoV-229E mainly cause asymptomatic or mild respiratory and gastrointestinal infections, they have been circulating in humans since they were recognized, and accounting for approximately 5–30% of common colds. Nonetheless, we have not treated hCoVs seriously until we witnessed the global epidemic caused by SARS-CoV and realized how devastating outcomes it brought to us [1]. To date, there have been three documented highly pathogenic and lethal hCoVs, namely SARS-CoV, MERS-CoV and SARS-CoV-2, because of their dreadful impacts on humans [4]. Unlike other hCoVs, SARS-CoV, MERS-CoV, and SARS-CoV-2 are prone to infect the lower respiratory tract, resulting in acute lung injury (ALI)/acute respiratory distress syndrome (ARDS), septic shock and multi-organ failure, with high case fatality ratio (CFR) [5]. As shown in Table 1, SARS-CoV first emerged in Foshan, China in November 2002 [16], and was subsequently transported to Hong Kong in February 2003, from where it spread globally [10]. The epidemic was finally contained in July 2003 as the transmission chain of SARS-CoV in Taiwan was interrupted [10, 17]. There were four instances of SARS reemergence that occurred chronologically in Singapore, Taipei, Guangdong and Beijing afterwards [10, 18]. No more infected human cases have been reported since May 2004. However, another deathful hCoV emerges only a decade later. MERS-CoV first occurred in April 2012 in Jordan [19] and has been causing persistent endemics in countries within and sporadically spreading to countries outside the Middle East regions [13]. The most recent laboratory-confirmed patients were reported by Riyadh on 28 March 2020 [20]. SARS-CoV-2 has emerged while humans continue to be threatened by MERS-CoV [21]. SARS-CoV-2 first occurred in Wuhan, China in December 2019 and it swiftly spread across China and has been aggressively infecting people globally. It was documented as a public health emergency of international concern and a pandemic on 30 January and 11 March 2020, respectively, making SARS-CoV-2 the first hCoV to cause a pandemic [6, 7]. Uniquely, the recent ongoing pandemic is accompanied by an infodemic, which has caused additional worldwide panic [22].

**Table 1:** The phylogenetic origin, crucial events and basic demographic information of SARS-CoV-2, SARS-CoV and MERS-CoV

	SARS-CoV-2[6–9]	SARS-CoV[10–12]	MERS-CoV[13–15]
**Genus**	Clade I, lineage B	Clade I, lineage B	Clade II, lineage C
**Length of nucleotides**	29.9 kilobases	29.75 kilobases	30.11 kilobases
**First emergence**	7 December 2019, Wuhan, China	16 November 2002, Foshan, China	4 April 2012, Zarqa, Jordan
**Virus identification**	January 2020	March 2003	June 2012
**Causative agent declaration**	January 2020	April 2003	September 2012
**Recent status**	Pandemic ongoing	Completely control	Sporadic continuous
**Number of infected cases**	Above 12.7 million^a^	8096	2553
**Male-to-female ratio**	1.27:1	1:1.13	1.78:1
**Number of attributed deaths**	Above 566 thousand^a^	774	876
**Number of viral Footprint**	213 countries or regions^a^	29 countries or regions	27 countries or regions
**Case fatal rate**	4.4%	9.6%	34.3%

*NA* Not available. ^a^ According to the data released by the WTO on 13, July, 2020

Although these deadly hCoVs have been posing dreadful threats to humans [23], there are no medicines or vaccines available, which highlights the urgent need to gain a deeper understanding of these lethal hCoVs and the illnesses caused by them, and the importance of fighting an infodemic simultaneously [24]. Thus, we aim to briefly summarize the cutting-edge knowledge and to provide an update of the major features of SARS-CoV, MERS-CoV, and SARS-CoV-2 in terms of animal hosts, morphology and genome organization, cellular entry and viral transmission, and cytokine and chemokine responses. We have included the predominant characteristics of illnesses caused by these hCoVs with respect to demographic characteristics, incubation period and clinical manifestations, laboratory tests, imaging performance, and pulmonary pathology.

## Traits of SARS-CoV, MERS-CoV, and SARS-CoV-2

### Animal hosts

**Fig. 1:**
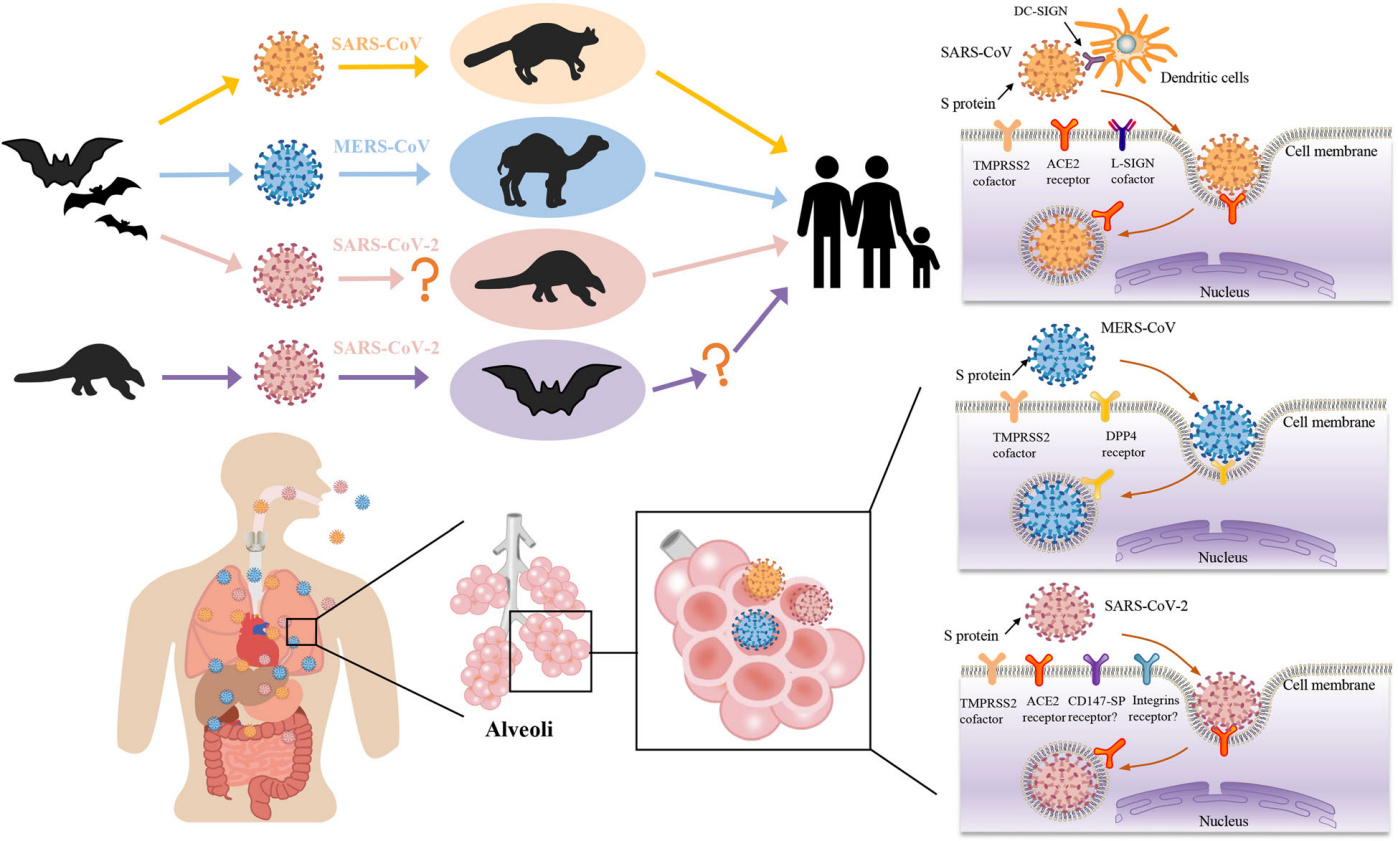
The potential animal hosts, biodistribution and host cell receptors of SARS-CoV, MERS-CoV and SARS-CoV-2

The potential animal hosts of SARS-CoV, MERS-CoV, and SARS-CoV-2 were summarized *in* Fig. 1. Notably, the outbreaks of these hCoVs are related to interactions between humans and animals, especially, both SARS-CoV and SARS-CoV-2 emerge from wet markets in China. Considering early SARS patients were associated with wild animal markets in Guangdong, SARS-CoV was considered to emerge from wild animals (included palm civets) which were sold in these markets [16]. Subsequently, a strain of CoV shared highly homological similarity to SARS-CoV (99.8%) was isolated from palm civets from wild animal markets, thus palm civets-derived CoVs were believed to be able to switch their hosts to human, causing the human-to-human transmission [25]. Additionally, the phenomenon that some SARS patients (3/4) had had a clear contact history with palm civets during the sporadic occurred in Guangdong was noticed, thereby epidemiologically supporting the previous assumption that palm civets act as important animal hosts of SARS-CoV [26]. Thus, it is reasonable to believe that palm civets were important intermediate hosts for SARS-CoV based on this strong evidence. Subsequently, scholars found that palm civets on farms are largely free from SARS-CoV infection while approximately 80% of the civets sold in an animal market were serologically positive (high level of SARS-CoV antibody), indicating that no widespread infection in wild civets [27]. Thus, palm civets became the intermediate hosts of SARS-CoV probably by getting infected during trade activities in or before reaching these wet markets [27, 28]. Afterwards, a strain of SARS-like-CoV was isolated from Chinese horseshoe bats, sharing 88–92% genomic identity to CoVs from humans or civet cats, strongly indicating that bats are natural hosts for SARS-CoV [29]. MERS-CoV is also believed to originate from bats [30]. The RNA fragment obtained by PCR amplification of nucleic acid isolated from bat stool shared 100% nucleotide identity with MERS-CoV from an infected case living in the same area, indicating bats were probably the source for MERS-CoV [31]. Then, a bat-CoV was demonstrated to hold a close phylogenetic relationship with MERS-CoV [32]. Subsequently, the ability of replicating in bats without generating symptoms of MERS patients was demonstrated, suggesting that bats were ideal reservoirs for MERS-CoV [33]. The intermediate reservoir role of dromedary camels for MERS-CoV was supported by abundant evidence [34]. Two virological studies illustrated that MERS-CoV was circulating in dromedary camels and indicated potential cross-infection between them and humans. The high genomic identity of MERS-CoV isolated from dromedary camels and humans was subsequently documented (99.2–99.5%) [35, 36]. Afterward, the reservoir possibility and natural host-to-human transmission role of dromedary camels were confirmed by several serological studies [37]. The origin of SARS-CoV-2 is more sophisticated. Similar to SARS-CoV, the emergence of SARS-CoV-2 was considered to be associated with trade activities in a wet market in Wuhan [21]. Researchers found that SARS-CoV-2 and BatCoV RaTG13 (a bat-CoV) were genetically similar and assumed that bats might be the natural reservoirs for SARS-CoV-2 [21]. Another study demonstrated that the similarity in genome between SARS-CoV-2 and the CoV isolated from pangolin (pangolin-CoV) was high but lower than that between SARS-CoV-2 and RaTG13 (91.02% vs. 96.2%) [38]. These findings were echoed by two other research, in which their genomic similarity are 90.03 and 92.4%, respectively [39, 40]. Scholars also analyzed the phylogenetic relationships among these CoVs, noticing that RaTG13 and SARS-CoV-2 were grouped together, and pangolin-CoV was their closest common ancestor. Taken together, Zhang et al. suggested that pangolin-CoV is another closely related kin of SARS-CoV-2, and pangolins rather than bats might be the natural reservoirs for SARS-CoV-2 and RaTG13 [38]. Although RaTG13 and SARS-CoV-2 share the highest homology regarding the overall genomic sequence, SARS-CoV-2 exhibits the highest sequence similarity (97.4%) to pangolin-CoV in terms of receptor-binding domain (RBD), however, RBD sequence similarity between RaTG13 and SARS-CoV-2 is far less (89.2%). More notably, six key RBD residues of SARS-CoV-2 and pangolin-CoV are completely identical while only one amino acid is the same between RaTG13 and SARS-CoV-2 regarding these six residues [21]. These findings rendered the issue that which one is the natural reservoir remains controversial, nonetheless, it is agreed that there exists other animals acting as intermediate hosts [41]. A study speculated snakes are probably the intermediate hosts because a similar synonymous codon usage bias was found among SARS-CoV-2, a bat-derived SARS-like-CoV, and snakes [42]. However, their research is far from enough to make such a conclusion. Notably, there were several shortages in their study as described by Li J and colleagues [43]. More importantly, close relative synonymous codon usage alone is inadequate and cannot be used as reliable evidence to assume that snakes might serve as intermediate hosts for SARS-CoV-2.

### Morphology and genome organization

Transmission electron microscopy images show that CoVs are spherical-shape viruses with spike proteins projecting from the virion surface, leaving themselves resemble solar crowns, therefore being termed “coronaviruses” [44]. Among RNA viruses, the genomic size of CoVs is only smaller than that of planarian secretory cell nidovirus (41 kilobases [kb]), ranging from 26 to 32 kb [45, 46]. Of these deadly hCoVs, MERS-CoV has the largest genomic size (approximately 30.11 kb), followed by SARS-CoV-2 and SARS-CoV, around 29.9 kb and 29.75 kb, respectively [47]. SARS-CoV-2 shares approximately 79.5% genomic homology with SARS-CoV while only about 50% similarity with MERS-CoV, indicating that SARS-CoV is closer to SARS-CoV [41]. These hCoVs all possess the typical genomic structure of betacoronaviruses, consisting of 5′ methylated caps and 3′ polyadenylated tails [21, 48]. The 3′-terminal region (one-third of the genome) is responsible for encoding structural proteins, namely spike protein, envelope protein, membrane protein, and nucleocapsid protein, which are critical for viral life cycle. The 5′-terminal region (two-thirds) is the non-structural protein coding region, comprising significant genes, which are essential for viral replication [48, 49]. Genomic knowledge of hCoVs promotes the understanding of the origin and pathogenesis (host immune response and viral virulence and transmission mode) of hCoVs, thus, a better understanding of viral genomic information is crucial for combating the outbreaks of hCoVs by setting up precise strategies, such as developing diagnostic systems, potential drugs and vaccine candidates promptly [50].

### Cellular entry and viral transmission

The spike protein not only acts as one of the requisite structural proteins of CoVs, but it also plays significant roles in the interaction between CoVs and host cells [51]. Structurally, spike protein consists of S1 and S2 subunit [51]. The RBD in the S1 subunit is responsible for viruses binding to host receptors and the S2 subunit is in charge of the fusion between viruses and host membranes, inducing the viral genome penetrates into host cells cytoplasm [52]. SARS-CoV, MERS-CoV and SARS-CoV-2 enter host cells are mediated by binding the receptor-binding domain to functional receptors on the host cell surface [53]. The angiotensin-converting enzyme 2 (ACE2) is the dominant host receptor of SARS-CoV [54]. DC-SIGN (CD209) and L-SIGN (CD209L) also function as co-receptors for SARS-CoV [55]. In contrast to ACE2, binding to DC-SIGN does not lead to SARS-CoV infection in dendritic cells but greatly enhances viral infection and dissemination. L-SIGN is also treated as an alternative receptor for SARS-CoV because L-SIGN can mediate cellular entry of SARS-CoV by binding to its spike protein [28].. The dipeptidyl peptidase 4 (DPP4, also termed CD26), is the cellular receptor for MERS-CoV [56]. Similarly, SARS-CoV-2 enters host cells by binding its spike protein to ACE2 [52, 53]. Importantly, ACE2 has a higher affinity to SARS-CoV-2 than to SARS-CoV [57]. Moreover, Christian et al. assumed that SARS-CoV-2 might alternatively use the integrins as cell receptors [58]. However, this assumption lacks strong experimental evidence. In contrast, stronger supporting evidence suggested that CD147-SP might be another entry route for SARS-CoV-2 [59]. Apart from the binding of spike proteins to functional receptors, the priming of spike proteins is also essential for hCoVs regarding cellular entry [60]. SARS-CoV, MERS-CoV and SARS-CoV-2 employ cellular serine protease TMPRSS2 and endosomal cysteine proteases cathepsin B/L for spike protein priming, which is essential for them to enter host cells [53, 61]. ACE2 has a vast biodistribution, including respiratory tract, gastrointestinal tract, heart, kidney and olfactory neuroepithelium [62, 63], besides these organs, DPP4 also expresses on liver, thymus, prostate and bone marrow [13], resulting in broad cellular and tissue tropisms of SARS-CoV, MERS-CoV, and SARS-CoV-2 [64–66]. Thus, these hCoVs can cause a wide range of symptoms, including respiratory manifestations and those beyond respiratory system to infected cases (described below), and their transmission routes are various as well. SARS-CoV-2 has the highest transmissibility, followed by SARS-CoV and MERS-CoV, of whom the basic reproductive number was projected to be 2–3.58, 1.7–1.9 and < 1, respectively [67]. With the upsurge in the number of SARS-CoV-2 patients worldwide, its median basic reproductive number was projected at 5.7 [68]. SARS-CoV was mainly transmitted by a close person-to-person contact through inhaling air droplets or by contacting with contaminated surfaces of devices [69, 70]. A major community outbreak occurred in Hong Kong indicated that SARS-CoV probably could be transmitted by airborne [71]. Given another outbreak was attributed to fecal contamination, feco-oral transmission should not be ignored [72]. As for MERS-CoV, humans can be infected by contacting with infected dromedary camels [30]. Similarly, human-to-human transmission is the major source of MERS-CoV transmission, however, MERS-CoV is not sustainably and frequently transmitted between humans [13]. Theoretically, MERS-CoV could also spread through contacting with stool, vomitus, urine, serum and cerebrospinal fluid of patients since MERS-CoV has been isolated from these samples [13]. Likewise, the key transmission path of SARS-CoV-2 is human-to-human transmission occurred in close contacts, predominantly spread by droplets and direct contact [73]. Besides, scarce and incomplete evidence indicates that maternal-fetal transmission of SARS-CoV-2 is likely possible but extremely rare [74]. Recently, researches showed that SARS-CoV-2 was detected in stool and its nucleocapsid protein was detected in gastrointestinal tissues, and live SARS-CoV-2 was cultivated from stool [66, 75, 76]. Notably, SARS-CoV-2 could be detected in sputum, urine, blood/serum, ocular surface, saliva and aerosol as well [66, 75, 77–79]. Although the detection or cultivation of SARS-CoV-2 in these specimens does not firmly mean that SARS-CoV-2 is transmitted by these samples, we should be careful when we are dealing with these samples.

### Cytokine and chemokine responses

**Fig. 2:**
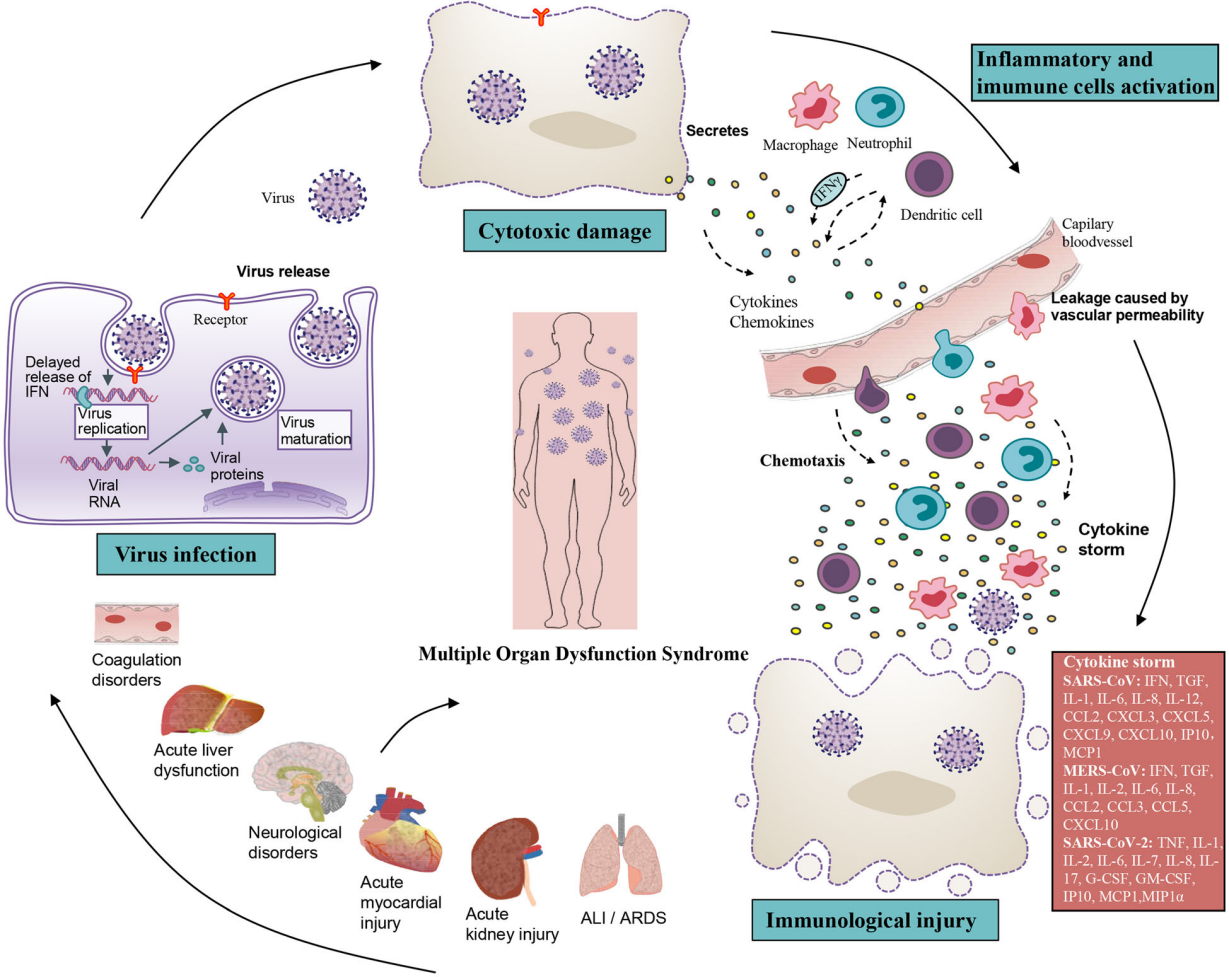
The mode by which lethal hCoVs lead cytotoxic damage (direct) and immunological injury (indirect) to host cells and cause multiple organ dysfunction syndrome

A moderate cytokine and chemokine response plays an indispensable role in the viral clearance and subsequent recovery while dysregulated response can bring devastating outcomes to infected cases [80]. A large number of immune cells, including macrophages, neutrophils, monocytes and lymphocytes, are migrated from bloodstream to infection site by the recruitment of hyperactive cytokines and chemokines, resulting in further release of high concentrations of various cytokines and chemokines and activation of immune cells, thereby underlying the basis of immune-mediated damages to hosts [72, 81]. The ways that SARS-CoV, MERS-CoV and SARS-CoV-2 cause histopathological injuries to infected cases are presented in Fig. 2. Briefly, these aforementioned hCoVs have been evolutionarily acquiring the ability to encode numerous proteins that allow them to evade from the host immune system, during which the delayed release of interferon plays a crucial role, then to attract and over-activate more inflammatory and immune cell, thereby inducing cytokine storm characterized by a massive secretion and hyper-activation of cytokines and chemokines until they have achieved sufficiently high titers [1, 80, 82], and finally to cause severe injury of infected tissues [81, 83]. Supporting evidence is abundant. The crucial roles of exaggerated pro-inflammatory cytokine and chemokine response resulted from infections of SARS-CoV and MERS-CoV in the exacerbation of SARS and MERS illnesses were firmly demonstrated [84]. Specifically, the delayed but excessive production of cytokines and chemokines was thought to be the induction of dysregulated innate immune response to SARS-CoV infection and poor outcomes on the basis of the fact that elevated serum levels and prolonged response of pro-inflammatory cytokines and chemokines were observed in SARS patients and were associated with the severity of SARS-CoV infection [28, 81]. Similar phenomena have been observed in patients infected by MERS-CoV, especially those who were severely infected, among whom numerous cytokines and chemokines were excessively activated, massive inflammatory and immune cells were promptly attracted and infiltrated in infected tissues, resulting in severe immunological injuries or even death [81, 85]. Likewise, the positive correlation between high pro-inflammatory cytokines and chemokines profile and the severity and outcomes of COVID-19 patients has been solidly confirmed, which indicates that SARS-CoV-2 infection also leads to hypercytokinemia or cytokine storm, by which ALI or ARDS and extrapulmonary multiple-organ failure or even death occur in infected cases [84, 86]. Similar to SARS, elevated levels of type 2 cytokines were also observed in COVID-19 [87, 88]. Although type 2 cytokines have anti-inflammation properties, and the expression of ACE2 was inhibited by them, surprisingly, they did not generate obvious benefits. This might be because type 2 cytokines simultaneously upregulated TMPRSS2 expression, which greatly negates their potential protective effects [89]. Overall, dysregulated cytokines and chemokines are associated with the progression and prognosis of infections caused by these hCoVs. Hence, interventions with these aberrant cytokines and chemokines might be promising for the managements of hCoVs-related diseases. Recently, many researchers are focused on the application of cytokine-based interventions, including immune inhibitors (such as inhibitors of IL-6, IFN-γ and TNF-α) in the therapy of COVID-19, and some of these inhibitors showed enthusiastic results (such as IL-6 inhibitor, siltuximab) [90, 91], however, more studies are needed to further investigate the therapeutic effects of these inhibitors.

## Features of SARS, MERS and COVID-19

### Demographic characteristics

The majority of SARS cases were from China, Canada and Singapore, among which, cases from China mainland presented the largest proportion, followed by that from Hong Kong and Taiwan [11]. There was a female predominance (53% vs. 47%) [11]. Adult patients presented approximately 93% of infected cases while only 7% cases were children. The mean age was 39.9 years, with a range of 1 to 91 years [12]. Saudi Arabia population presented nearly 80% of MERS cases and around 91.0% of deaths, with a roughly CFR of 37.1%, which nearly quadrupled that of SARS [14]. There was a sexual predisposition to male, while male patients presented 64% of total patients, female patients only accounted for 36%. The percentage of patients in adults was overwhelmingly surpassed that in children, which were 98 and 2%, respectively. The median age was 50 years, with a range of 1 to 94 years [13, 15]. As for COVID-19, the numbers of infected cases and deaths keep increasing rapidly every day. As shown in Table 2, the numbers of patients, deaths and affected countries or regions have far exceeded those of SARS-CoV and MERS-CoV [8]. Similar to MERS, more patients were male, while male patients presented 55.9% of total cases, female patients only accounted for 44.1% [9]. Wu and colleagues showed that the age of most Chinese patients (38,680/44672) ranged from 30 to 79 years [101]. The median age ranged from 34 to 63 years old according to different research [102–105]. Similarly, SARS-CoV-2 was less likely to infect children and adolescents [106]. Wu et al. reported that only 2.2% of 44,672 confirmed cases occurred in persons aged younger than 19 [101]; among 149,082 cases reported by American CDC, only 1.7% patients were aged younger than 18 [107]. Notably, among 1099 Chinese patients, only 10 cases (0.9%) were younger than 15-year-old [103]. In contrast, the elderly not only are vulnerable to SARS-CoV-2, but they also are more susceptible to be severely infected by this hCoV; moreover, the senior population has higher CFR as well [101, 108, 109]. Although the reasons for the differences between young and senior generations are yet unclear, different expression levels of ACE2 and TMPRSS2 and different immune systems between them might be the possible explanations [106, 110], and the elderly have more comorbidities and senile immune systems might be the predominant factors.

**Table 2:** Clinical characteristics and laboratory findings of COVID-19, SARS and MERS patients

	COVID-19 [94, 95, 98]	SARS [25, 97–99]	MERS [92, 93, 96, 98, 100]
**Signs and symptoms**			
Fever	56–99%	99–100%	81.7–100%
Fatigue	18–55%	31.2%	NA
Cough	39–81%	29.0–74.3%	75–85%
Sore throat	5–17%	11.0–23.2%	14
Dyspnea	12–41%	40–42%	72%
Myalgia	18–55%	49.3–60.9%	38
Diarrhea	3–17%	20–25%	26
Headache	4–23%	15.0–55.8%	NA
**Complications**			
ARDS	18–30%	20%	20–30%
AKI	3%	6.7%	41–50%
**Laboratory findings**			
Leukopenia (< 4.0 × 10^9^/L)	26.8%	23–35%	14%
Lymphopenia (< 1.5 × 10^9^/L)	55.3%	68–85%	32%
Thrombocytopenia (< 150 × 10^9^/L)	11.5%	40–45%	36%
Elevated LDH	55.5%	50–71%	48%
Elevated AST	17.9%	20–30%	14%
Elevated ALT	16.0%	20–30%	11%

*LDH* Lactate dehydrogenase, *AST* Aspartate aminotransferase, *ALT* Alanine aminotransferase, *NA* Not available

### Incubation period and clinical manifestations

During the incubation period, hCoVs will not cause overt clinical symptoms, but the knowledge of viral incubation period has significant applications in disease surveillance, prevention and control [111]. The median incubation period of SARS-CoV is 4 days (95% CI 3.6–4.4) [111] and a longer one with >10 days was only observed in a small proportion of cases [112]. The median incubation period of MERS-CoV was 5.2 days (95% CI 1.9–14.7) and the period could be longer in immunocompromised patients or those with comorbidities as well [13, 113]. The longest incubation period was 20 days and observed in a female who had received hematopoietic stem cell therapy after the recurrence of diffuse large B-cell lymphoma [113], which indicated that it is significant to evaluate the overall status of every suspicious or confirmed case when surveillance, prevention and control of infectious disease are carried out. While the incubation period of SARS-CoV-2 is yet unclear, it is estimated to be consistent with that of SARS-CoV and MERS-CoV. The estimated median incubation period was consistent (4 days) but their interquartile ranges were various in three independent investigations (2–7 days, 3–6 days and 2.3–4.3 days, respectively) [103, 114, 115]. It could be longer, as reported by Lauer and colleagues, the estimate was 5.1 days (95% CI, 4.5–5.8 days) [116]. Linton and colleagues showed that the mean incubation period was 5.0 days (95% CI 4.2–6.0 days). This estimate was in line with a meta-analysis, showing that the pooled mean incubation period was 5.08 days (95% CI 4.77–5.39 days) [117, 118]. Similarly, a longer estimate was also reported, which was 6.4 days (95% CI 5.6–7.7 days) [119]. Recently, a pairwise comparison showed that there is no statistically significant difference in the incubation period among these three hCoVs [120]. In contrast, many pathological abnormalities emerge subsequence to hypercytokinemia or cytokine storm, including weakened stabilization of endothelial cell to cell interactions, damaged integrity of vascular barrier and capillary, diffused damage of alveolus, and multiple organs dysfunction [121], resulting in the onset of acute respiratory infections with systematic disorders after the incubation period [10, 13, 103, 122]. Hence, as shown in Table 2*,* although the clinical manifestations of SARS, MERS and COVID-19 are pretty similar, including fever (≥38.0 °C), cough, sore throat, dyspnea, headache, myalgia or fatigue, and diarrhea [99, 123, 124], patients would probably present a wide range of symptoms. The disease course of SARS is usually divided into two periods, namely the early period (1–7 days) and progress period (10–14 days). Usually, in the early period, SARS patients (except those started with dry cough) were additionally suffered from nonproductive cough 3 to 7 days after the occurrence of early symptoms; in progress period, patients’ conditions obviously deteriorated, and some (10–20%) ended with fatal outcome [10, 99]. MERS patients are usually manifested as severe respiratory infection once symptoms appeared and patients’ condition progresses rapidly [13, 100]. Significantly, acute kidney injury (AKI) is one atypical symptom of MERS patients, which promptly occurs in more than half of MERS cases after the viral incubation period (around a week) [92]. Compared with SARS patients, medical comorbidities include diabetes, hypertension, cardiovascular diseases, chronic renal failure and chronic pulmonary disease are more common in MERS patients, which partially account for the high CFR [93]. The clinical manifestations of COVID-19 are predominantly shared by SARS and MERS [94, 103]. Apart from diarrhea, other gastrointestinal symptoms, including nausea and vomiting are common in COVID-19 patients as well [125]. COVID-19 patients predominantly present with mild symptoms, but those with comorbidities have worse clinical outcomes [126]. Although the previous CFR of COVID-19 was far less than that of its counterparts, the CFR of COVID-19 has gradually increased as the mounting number of deaths worldwide [8, 11, 14]. Additionally, there are asymptomatic patients as well. Although these cases were asymptomatic, they can disseminate hCoVs, thereby posing a great challenge to infection control. Thus, it is of great significance to better understand the aspect of these hCoVs, however, asymptomatic case rate is difficult to estimate. To date, the estimated asymptomatic infections incidence various in different research. Worse still, these cases reported in these investigations only presented the tip of the iceberg, and the true rates remain unclear [118, 127–129].

### Laboratory tests

Molecular tests such as polymerase chain reaction using viral RNA extracted from clinical samples have become the standard and primary diagnostic test of SARS, MERS and COVID-19 due to its high sensitivity, specificity and simplicity [122, 130]. However, the sensitivity of serology tests such as antibody detection was generally lower than that of molecular tests and antibody detection was predominantly used in retrospective diagnosis for SARS and MERS [93]. Similarly, the slow plasma antibody responses to SARS-CoV-2 were confirmed, however, serological assay remains significant for the diagnosis and management of COVID-19 because the combination of antibody test greatly increased the sensitivity of viral RNA detection in the diagnosis of SARS-CoV-2 [131]. As presented in Table 2, the laboratory findings of SARS, MERS and COVID-19 patients are greatly similar, of whom the commonest abnormal laboratory findings are lymphocytopenia and thrombocytopenia. In addition, the serum levels of lactate dehydrogenase, aspartate aminotransferase, alanine aminotransferase and C-reactive protein are significantly elevated [95–97]. Coagulation disorders characterized by elevated D-dimer level and prolonged prothrombin time are common, especially in severe patients [98]. Meanwhile, elevated level of creatine kinase and serum creatinine in diverse degrees were commonly found in some patients, especially in MERS patients [95–97].

### Chest radiology

The imaging performance of viral pneumonia is almost overlapping, however, some specific differences exist as well. Although the chest X-ray/CT performance of pneumonia caused by SARS-CoV, MERE-CoV, and SARS-CoV-2 are similar, chest CT is preferred due to its high resolution, sensitivity and efficacy. As shown in Table 3, the commonest chest radiological performance of SARS, MERS and COVID-19 patients is multifocal or mixed ground-glass opacities, or crazy paving pattern in some cases, followed by consolidation, smooth or irregular interlobular septal thickening and air bronchogram [132–137]. Pleural effusion is rare or only occurs in severe SARS and COVID-19 patients, while it is common in MERS cases (roughly 33–50%). Pneumothorax and centrilobular nodules can be detected only in a few patients, whilst cavitation and lymphadenopathy are both rare or absent. Notably, in most COVID-19 cases, both lungs (multiple lobes, especially the lower lobes are involved) are simultaneously infected by SARS-CoV-2, exhibiting peripheral distribution on chest CT images, nonetheless, in the initial period of SARS and MERS, lungs are more commonly involved in unilateral or unifocal than multifocal involvement. Recently, PET/CT has been developed to image and measure lung inflammation [138]. A COVID-19 case series research demonstrated that pulmonary peripheral ground-glass opacities and lung consolidations are characterized by a high ^18^F-FDG uptake and lymph node involvement was supported by PET/CT examination [139]. Lung ultrasound has recently become a reliable and convenient technique, playing an auxiliary role in diagnosing and evaluating the severity of respiratory diseases, such as interstitial lung disease, ARDS, acute pulmonary edema, and pneumonia pleural effusion, pneumothorax, atelectasis, and pulmonary embolism [140, 141]. Besides, lung ultrasound contributes to the diagnosis and severity assessment of COVID-19. The main ultrasonic signs of COVID-19 are bilateral thickening and irregular pleural line; various patterns of B-line including focal, multifocal and confluent; various patterns of consolidations including small and translobar with or without mobile air bronchograms. Besides, pleural effusion can be detected in some patients as well, but it is rare [142, 143].

**Table 3:** Chest X-ray/CT features of COVID-19, SARS and MERS patients

	COVID-19 [132, 133]	SARS [134, 135]	MERS [136, 137]
**Image performance**	Bilateral, multifocal, peripheral distribution	Unilateral, focal; unilateral, multifocal; bilateral; peripheral distribution	Bilateral, multifocal; isolated unilateral; peripheral distribution
**Normal radiography**	19.90%	18.40%	20.00%
**Abnormal radiography**			
Ground-glass opacities	68.92%	68.48%	86.36%
Crazy paving pattern	8.56%	46.27%	26.67%
Consolidation	26.64%	65.65%	50.00%
Interlobular septal thickening	34.54%	55.22%	40.91%
Air bronchogram	34.54%	37.04%	NA
Pleural effusion	3.57%	17.31%	54.55%
Pneumothorax	Rare	9.62%	Rare
Centrilobular nodules	Not seen	Not seen	Not seen
Cavitation	Not seen	Not seen	Not seen
Lymphadenopathy	6.00%	Not seen	Not seen

*NA* not available

### Pulmonary pathology

As shown in Table 4, pulmonary histopathological abnormalities of SARS, MERS and COVID-19 cases are non-specific. These changes result from direct viral cytotoxic and immunopathogenic effects. They are mainly characterized by diffuse alveolar damage (DAD), which includes two categories, namely acute exudative DAD and proliferative DAD. Several SARS autopsy research showed that SARS-CoV could damage multiple tissues, however, the major histopathology involves lungs [55, 150, 151]. Different traits of DAD were observed during different disease stages [152]. Specifically, acute exudative DAD is the predominant pulmonary pathology finding of early period SARS. Besides, proliferative DAD was additionally observed in the progress period. Notably, with the extension of illness duration (over 2–3 weeks), the organizing and proliferative features of DAD became obvious while the exudative traits of DAD were rarely seen [55, 153, 154]. Similarly, DAD is the predominant pathological feature of MERS based on autopsy investigations of MERS [13, 155, 156]. Besides, focal hemorrhagic necrotizing pneumonia was also observed in MERS cases [156]. Considering autopsy studies were rarely performed, some experiments were carried out, of which the findings were consistent with that observed in humans [157, 158]. The major pulmonary histological performance of COVID-19 greatly resembled those of SARS and MERS, but differences exist as well. Similar to SARS, the microscopic manifestations of COVID-19 are different in different stages of illness [55, 144, 145]. The major pathology manifestations of COVID-19 include bilateral DAD as well as interstitial inflammation and fibrosis [146, 147]. Pleural lesions, mucous plugs and inflammatory cell infiltration were observed [148]. Whether hyaline membrane formation in infected lungs remains controversial in different research. Tian and colleagues compared the differences of histological features between early-stage COVID-19 and advanced-stage COVID-19 and declared that the formation of hyaline membrane might be a pathological characteristic of advanced-stage COVID-19 [144, 145]. Notably, intravascular microthrombi were found in patients with SARS and COVID-19 [55, 149, 153], and the combination of DAD and thrombosis contributed to the rapid deterioration of clinical conditions in severe COVID-19 cases [152].

**Table 4:** Pulmonary pathology of COVID-19, SARS, and MERS

	COVID-19[144–149]	SARS[55, 150–154]	MERS [13, 155–158]
Gross examination	Diffuse congestions with partly hemorrhagic necrosis	Edematous lungs with diffuse congestion, enlarge pulmonary hila lymph nodes, irregular and patchy consolidation areas	Edematous lungs with consolidation
Microscopic manifestation	Main abnormalities: 1. Early-stage: exudative DAD without hyaline membrane formation; 2. Advanced-stage: DAD with hyaline membrane formation; Others: pleural effusion and adhesion, mucous plugs formation, macrophages, neutrophils and lymphocytes infiltration; microvascular injury (microthrombi)	Main abnormalities: 1. Acute-period: acute exudative DAD (extensive edema and hyaline membrane formation, alveolar epithelial cells impairment, alveoli collapse, and fibrous tissue in alveolar spaces); 2. Progress-period: Combination of acute exudative DAD and proliferative DAD (fibrinous interstitial and airspace and hyperplastic pneumocytes); Others: intravascular microthrombi	Main abnormalities: Acute exudative DAD with focal hemorrhagic necrotizing pneumonia (dispersed necrotic debris); Others: NA
Superinfection	Bacteria	Bacteria, fungi, viruses	NA

*NA* not available

## Conclusion

Although there are many similarities among SARS-CoV, MERS-CoV, and SARS-CoV-2 and severe illnesses cause by them, these lethal hCoVs and illnesses are characterized by distinctive traits. The periodical emergence of highly pathogenic hCoV has been sustainably posing heavy burden and threat to humans. Though some drugs were thought to be promising candidates for COVID-19 therapy, they were experimentally labeled as inefficient because of lacking therapeutic effects with statistical importance or reasonably satisfactory clinical outcomes [159], thus, approval medicines remain absent so far, and vaccines either. What should bear in mind is that MERS-CoV remains circulates [13] and the number of SARS-CoV-2 infected cases and deaths continues climbing quickly and the fast spread of SARS-CoV-2 attributed infodemic has been causing unnecessary panic globally. Thus, more research are urgently needed to unveil the secrets of these deadly hCoVs and related infections, especially in developing specific medicine and vaccines, and effective interventions should be prepared in case of the emerge or reemerge of hCoVs in the future as well, thereby minimizing the burden and threat resulted from the infections and spreads of hCoVs.

## Data Availability

Not applicable.

## Abbreviations

CoVs: Coronaviruses
hCoVs: Human coronaviruses;
SARS: Severe Acute Respiratory Syndrome
MERS: Middle East Respiratory Syndrome
COVID-19: Coronavirus Disease 2019
ALI: Acute lung injury
ARDS: Acute respiratory distress syndrome
AKI: Acute kidney injury
CFR: Case fatality ratio
WHO: World Health Organization
ACE2: Angiotensin-converting enzyme 2
DPP4: Dipeptidyl peptidase 4
DAD: Diffuse alveolar damage.
